# Distribution of *Clba* Gene and Its Correlation With Antimicrobial Resistance Patterns in MDR *E. coli* From Diverse Host Groups

**DOI:** 10.1002/mbo3.70142

**Published:** 2025-11-10

**Authors:** Soma Kanta Baral, Govardhan Joshi, Indira Parajuli, Krishna Das Manandhar, Pramod Poudel

**Affiliations:** ^1^ Central Department of Biotechnology Tribhuvan University Kathmandu Nepal; ^2^ Department of Laboratory Medicine Manmohan Memorial Institute of Health Sciences Kathmandu Nepal; ^3^ Central Department of Environmental Science Tribhuvan University Kathmandu Nepal

**Keywords:** antimicrobial resistance, cancer, clbA, clinical infection, colibactin, MDR *E. coli*

## Abstract

Multidrug‐resistant (*MDR*) *Escherichia coli* (*E. coli*) represents a significant public health concern, particularly when harboring virulence genes such as clbA, which encodes the genotoxin colibactin. This study assessed the distribution of the clbA gene among MDR *E. coli* isolates from normal individuals, cancer patients, and clinical patients, and examined its association with antimicrobial resistance patterns. A total of 115 MDR *E. coli* isolates were collected from January to December 2024 at two healthcare centers in Nepal. The clbA gene was detected in 13.0% (15/115) of isolates, with a significantly higher prevalence in clinical patients (25.0%) compared to cancer patients (8.6%) and normal individuals (5.0%) (*p* = 0.0105). clbA‐positive isolates exhibited markedly increased resistance to critical antibiotics, including imipenem (100% vs. 15.0%, *p* = 0.003), meropenem (100% vs. 12.5%, *p* = 0.001), and amikacin (100% vs. 10.0%, *p* = 0.050), compared to clbA‐negative strains. These findings suggest that the presence of the colibactin‐encoding clbA gene in MDR *E. coli* is linked to heightened antimicrobial resistance, especially in clinical settings, underscoring the need for targeted molecular surveillance and infection control strategies.

## Graphical abstract

The clbA gene, encoding the genotoxin colibactin, was present in 13.0% of Multidrug‐resistant (MDR) *E. coli* isolates, with a significantly higher prevalence observed in clinical patients (25.0%) compared to other host groups. clbA‐positive isolates demonstrated a markedly enhanced resistance profile, including 100% resistance to critical antibiotics such as meropenem, imipenem, and amikacin. These findings underscore the dual threat posed by clbA ‐positive MDR *E. coli*: genotoxicity and enhanced antimicrobial resistance, warranting targeted surveillance in clinical settings.

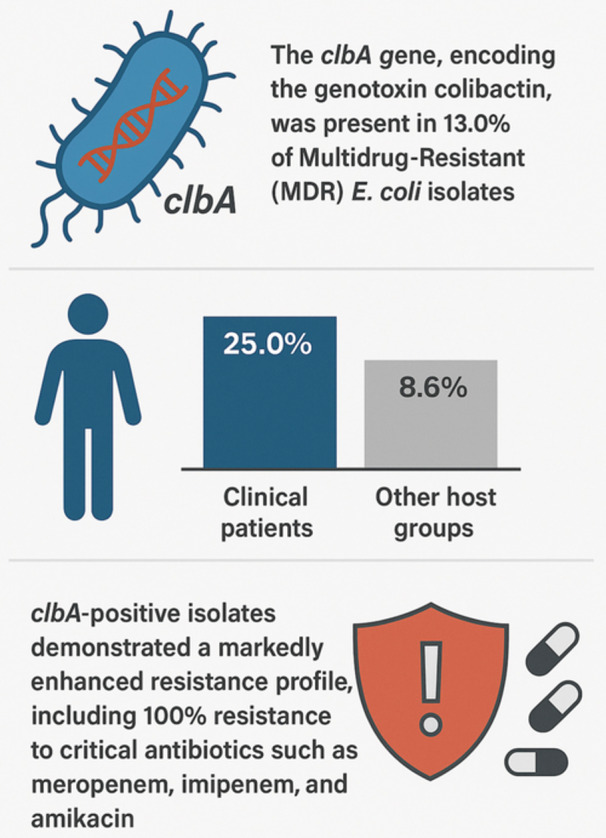

## 1 Introduction

Colorectal cancer (CRC) is a multifactorial disease influenced by genetic predisposition, environmental exposure, and microbial dysbiosis. Globally, CRC ranks as the third most commonly diagnosed cancer and the second leading cause of cancer‐related deaths, accounting for approximately 10% of new cancer cases and 9% of all cancer deaths worldwide (Sung et al. 2021). A substantial portion of this burden about 95% of disability adjusted life years (DALYs) is attributed to premature mortality, with only 5% resulting from disability (GDB 2019). Consequently, timely detection and effective prevention strategies are essential for mitigating disease impact and improving patient outcomes.

Recent studies have highlighted a strong association between gut microbiota and CRC development. Among these microbes, *E. coli* strains harboring the polyketide synthase (*pks)* genomic island, particularly those carrying the colibactin synthesis‐associated protein A (*clbA)* gene, are of increasing concern due to their ability to produce colibactin, a genotoxin that induces DNA damage and promotes tumorigenesis (Pleguezuelos‐Manzano et al. 2020; Yang et al. 2024). The *clbA* gene encodes a phosphopantetheinyl transferase essential for initiating colibactin biosynthesis and serves as a reliable molecular marker for identifying colibactin‐producing *E. coli* strains (Velilla et al. 2023).

Pks⁺ *E. coli* strains have been detected in both CRC patients and healthy individuals, with fecal carriage rates ranging from 12% to 32% in the general population (Massip et al. 2019). Alarmingly, around 20%–22% of asymptomatic carriers harbor these potentially genotoxic strains, indicating a silent risk for CRC development over time (Zhao et al. 2014). Despite growing concern, the prevalence of *clbA*‐positive strains among multidrug‐resistant (MDR) *E. coli* remains insufficiently characterized. This gap is clinically significant, as MDR pathogens complicate treatment protocols, exacerbate inflammation, and may contribute to cancer progression particularly in immunocompromised hosts such as cancer patients (Dobrindt and Hacker 2001).

The detection of *clbA* in *E. coli* isolates from diverse sources including healthy individuals, cancer patients, and clinical infections raises important questions about the convergence of antibiotic resistance and microbial genotoxicity. This combination may enhance bacterial virulence, limit treatment options, and increase susceptibility to persistent infection and malignancy (Russo and Johnson 2003).

This study aims to investigate the prevalence and distribution of the colibactin‐encoding *clbA* gene among MDR *E. coli* isolates obtained from clinical, oncological, and community sources. By characterizing both the molecular features and antimicrobial resistance profiles of these isolates, the research seeks to clarify the potential role of *clbA*‐positive *E. coli* in colorectal carcinogenesis. Previous reports have highlighted the urgent need to understand not only the pathogenic potential of colibactin‐producing *E. coli* (CoPEC) but also how their resistance traits may impact therapeutic outcomes, especially in immunocompromised hosts such as cancer patients (Lopès et al. 2020; Arthur et al. 2012). However, most existing studies focus narrowly on intestinal carriage in CRC patients, with limited comparative insights across diverse host groups particularly in regions facing high antibiotic pressure and limited antimicrobial stewardship. By bridging this gap, our study provides valuable epidemiological evidence from multiple populations, potentially informing risk assessment, early detection, and targeted interventions in both oncology and infectious disease practices (Putze et al. 2009; Johnson et al. 2015).

## 2 Materials and Methods

### 2.1 Study Design and Sample Collection

A cross‐sectional study was conducted to detect the presence of the ***clbA*** gene among MDR *E. coli* isolates. A total of 245 non‐duplicate *E. coli* isolates were collected over a period of 1 year from three different sources:(i) clinical specimens (urine, blood, and wound swabs) from both outpatients and inpatients, (ii) stool samples from patients undergoing cancer treatment, and (iii) fecal samples from apparently healthy individuals in community settings.

### 2.2 Bacterial Identification and Antimicrobial Susceptibility Testing

#### 2.2.1 Isolation and Identification of *E. coli*

Clinical specimens (pus, sputum, and urine) were cultured on MacConkey, Nutrient, and Blood Agar, while stool samples were cultured only on MacConkey Agar, using standard aseptic techniques and incubated at 37°C for 18–24 h. Lactose‐fermenting colonies on MacConkey Agar were selected for further testing. Presumptive *E. coli* identification was based on colony morphology, Gram staining (Gram‐negative rods), and biochemical tests (indole‐positive, methyl red‐positive, Voges‐Proskauer‐negative, citrate‐negative, urease‐negative) with an A/A reaction on TSI without H₂S production (Jorgensen and Pfaller 2019; Tadesse et al. 2020).

#### 2.2.2 Identification of MDR *E. coli*

Antimicrobial susceptibility testing was performed using the Kirby‐Bauer disk diffusion method on Mueller‐Hinton Agar following CLSI guidelines (Clinical and Laboratory Standards Institute 2023). Bacterial suspensions were adjusted to a 0.5 McFarland standard and inoculated onto MHA. Antibiotic discs were applied, and plates were incubated at 37°C for 18–24 h. Isolates resistant to ≥ 1 agent in ≥ 3 antimicrobial classes were classified as MDR (Magiorakos et al. 2012). Intermediate susceptibility results were excluded from MDR classification. Only isolates categorized as resistant were considered MDR; intermediate susceptibility results were excluded. The following antibiotics were tested: Amoxycillin (25 µg), Cefoxitin (30 µg), Cefpodoxime (10 µg), Ceftazidime (30 µg), Cefotaxime (30 µg), Cefixime (5 µg), Cefepime (30 µg), Cotrimoxazole (25 µg), Imipenem (10 µg), Meropenem (10 µg), Tetracycline (30 µg), Gentamycin(30 µg), Amikacin (30 µg), Ciprofloxicin (5 µg), Levofloxacin (5 µg), and Aztreonam (30 µg).

#### 2.2.3 DNA Extraction by Heat Method

Genomic DNA was extracted using the heat lysis method as described by Capobianco et al. (Capobianco et al. 2020). Briefly, a single colony of *E. coli* was suspended in 100 µL of sterile distilled water and heated at 95°C for 10 min. The lysate was immediately cooled on ice and centrifuged at 12,000 rpm for 5 min. The supernatant containing DNA was stored at −20°C for PCR analysis.

#### 2.2.4 Polymerase Chain Reaction (PCR) Detection of the *clba* Gene

**Figure 1:**
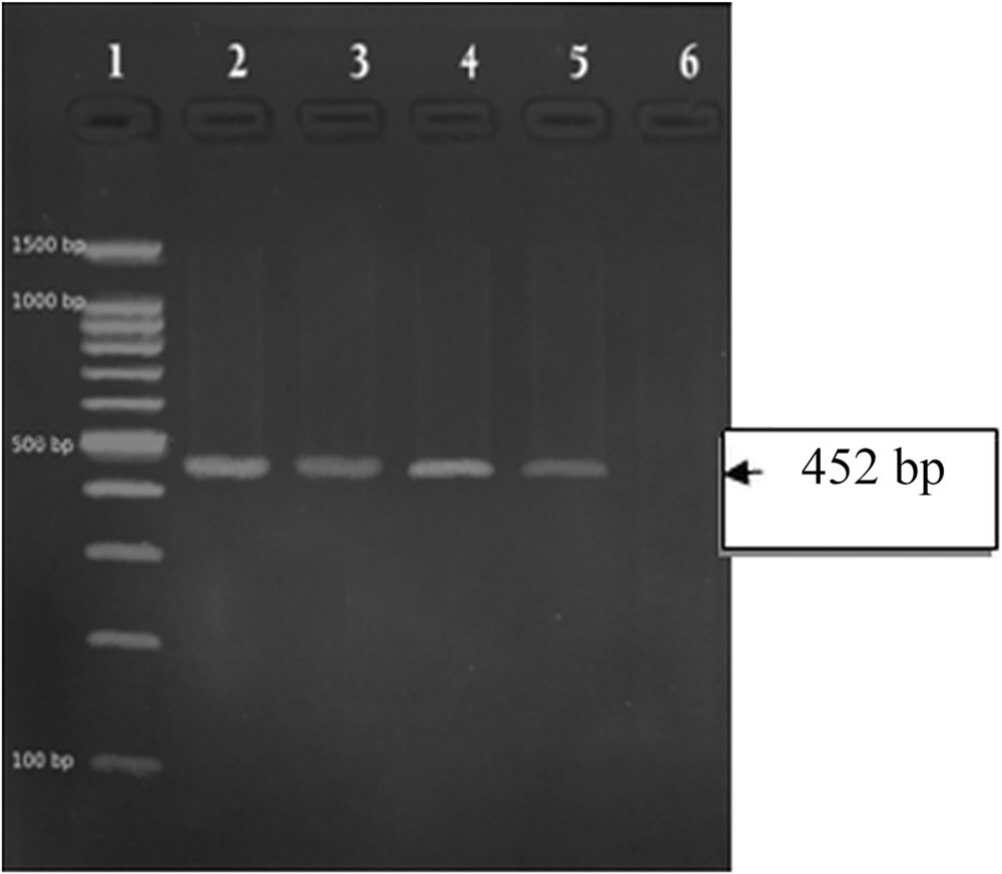
Amplicon of clb gene from MDR *E coli*.

The PCR amplification of the *clbA* gene was performed as previously described (Johnson et al. 2008), with minor modifications. The reaction was carried out in a 20 µL reaction mixture containing 12 µL of 10× Master Mix, 1.0 µL each of forward primer (clbA‐F: CAG ATA CAC AGA TAC CAT TCA) and reverse primer (clbA‐R: CTA GAT TAT CCG TGG CGA TTC), 2.0 µL of DNA template, and 4 µL of nuclease‐free water. The thermal cycling conditions were as follows: initial denaturation at 94°C for 15 min; 30 cycles of denaturation at 95°C for 30 s, annealing at 60°C for 30 s, and extension at 72°C for 90 s; followed by a final extension at 72°C for 10 min. PCR products were electrophoresed on a 1.5% agarose gel at 100 V for 30 min and visualized under UV light using a gel documentation system, as shown in Figure 1.

### 2.3 Statistical Analysis

Data analysis was performed using IBM SPSS Statistics version 20. The prevalence of the *clbA* gene among MDR *E. coli* isolates was calculated as a percentage for each study group. Categorical variables, including the distribution of *clbA* and antibiotic resistance profiles across clinical, oncological, and community‐derived isolates, were compared using the Chi‐square (χ²) test. A *p*‐value of < 0.05 was considered statistically significant.

### 2.4 Ethical Consideration

This study received ethical clearance from the Institutional Review Committee (IRC) of the Manmohan Memorial Institute of Health Sciences (MMIHS), Kathmandu, Nepal (Approval No. NEHCO/IRC/080/208). Before enrollment, the study's aims and objectives were thoroughly explained to each participant. Written informed consent was obtained to confirm their voluntary involvement. All research activities adhered to ethical standards, with strict measures taken to maintain the confidentiality and privacy of participant information.

## 3 Results

### 3.1 Distribution of MDR *E. coli* Among Diverse Host Groups

Out of the 346 samples analyzed, *E. coli* was isolated from 245 (70.8%) samples. The highest rate of *E. coli* positivity was observed among patients (76.9%) and normal individuals (75.9%), whereas cancer individuals showed a comparatively lower positivity rate (57.0%). Notably, the prevalence of MDR *E. coli* isolates was highest among cancer individuals, with 61.4% of isolates exhibiting multidrug resistance. This rate was substantially higher than that observed in clinical patients (44.4%) and healthy individuals (40.8%), as presented in Table 1. Despite this apparent difference, the chi‐square test did not reveal a statistically significant association between subject group and MDR prevalence (χ² = 4.82, *p* = 0.090), although the *p*‐value suggests a trend towards higher MDR rates in cancer individuals. These findings indicate a potential increased burden of MDR *E. coli* among cancer patients, warranting further investigation with larger sample sizes to confirm this association. Tables 2 and 3.

**Table 1:** Distribution of *Escherichia coli* and MDR *E. coli* among diverse host groups.

Subject group	No. of samples	Positive for *E. coli*, *n* (%)	MDR *E. coli*, *n* (%)	*p* value
Patients	117	90 (76.9%)	40 (44.4%)	
Normal individuals	129	98 (75.9%)	40 (40.8%)	0.090
Cancer individuals	100	57 (57.0%)	35 (61.4%)	
**Total**	**346**	**245 (70.8%)**	**115 (46.9%)**	

**Table 2:** Distribution of *clbA* gene among MDR *E. coli* isolates from different subject groups.

Subject group	No. of isolates	*clbA*‐positive *n* (%)	*clbA*‐negative *n* (%)	χ² value	*p* value
Patients	40	10 (25.0%)	30 (75.0%)	**9.12**	**0.0105**
Normal individuals	40	2 (5.0%)	38 (95.0%)		
Cancer individuals	35	3 (8.6%)	32 (91.4%)		
**Total**	**115**	**15 (13.0%)**	**100 (87.0%)**		

**Table 3:** Resistance pattern of MDR *E. coli* isolates by clbA status.

Antibiotic	clbA‐negative MDR *E. coli* (*n* = 100) (%)	clbA‐positive MDR *E. coli* (*n* = 15) (%)	*p* value
Amoxycillin (AMX)	100.0	100.0	1.000
Cefoxitin (COX)	100.0	100.0	1.000
Cefixime (CX)	97.5	90.0	0.200
Cefpodoxime (CFM)	100.0	100.0	0.083
Ceftazidime (CAZ)	85.0	100.0	0.050
Cefotaxime (CTX)	80.0	100.0	0.050
Cefepime (CPM)	72.5	100.0	0.050
Aztreonam (AT)	52.5	90.0	0.107
Cotrimoxazole (COT)	65.0	100.0	0.107
Ciprofloxacin (CIP)	65.0	100.0	1.000
Levofloxacin (LE)	67.5	100.0	0.050
Tetracycline (TE)	67.5	100.0	0.050
Gentamycin (GEN)	30.0	100.0	1.000
Amikacin (AK)	10.0	100.0	0.050
Meropenem (MRP)	12.5	100.0	0.001
Imipenem (IPM)	15.0	100.0	0.003

### 3.2 Distribution of *Clba* Gene Among MDR *E. coli* Isolates From Different Subject Groups

Out of 115 MDR *E. coli* isolates, the *clbA* gene was detected in 15 isolates (13.0%). The distribution of *clbA*‐positive isolates varied significantly across the study groups. Among isolates from clinical patients, 10 out of 40 (25.0%) were *clbA*‐positive, compared to 3 out of 35 (8.6%) from cancer patients and 2 out of 40 (5.0%) from normal individuals. The remaining 100 isolates (87.0%) were *clbA*‐negative.

Statistical analysis using the Chi‐square test revealed a significant association between *clbA* gene presence and the source of the isolate (χ² = 9.12, *p* = 0.0105), indicating that MDR *E. coli* isolates from patients were significantly more likely to harbor the *clbA* gene compared to those from cancer or healthy individuals. These findings suggest a potential clinical relevance of colibactin‐producing *E. coli* strains, especially in patient‐derived infections, warranting further investigation into their role in pathogenesis and possible links to colorectal carcinogenesis.

The chi‐square (χ²) test was applied to evaluate associations between categorical variables, specifically the distribution of the *clbA* gene across host groups and the relationship between *clbA* status and antibiotic resistance patterns.

### 3.3 Association of Antibiotic Resistance With the *Clba* Gene

Among 115 MDR *E. coli* isolates analyzed, 15 (13.0%) were *clbA*‐positive and 100 (87.0%) were *clbA* negative. Antibiotic susceptibility testing revealed significantly higher resistance rates in *clbA* positive isolates across multiple drug classes.

Carbapenem resistance was notably elevated in *clbA* positive strains, with 100% resistance to both meropenem and imipenem, compared to 12.5% and 15.0% in *clbA* negative isolates (*p* = 0.001 and 0.003, respectively). Similarly, resistance to aminoglycosides such as amikacin (100% vs. 10.0%, *p* = 0.050) and gentamicin (100% vs. 30.0%, *p* = 1.000) was more prevalent among *clbA*‐positive isolates. Both groups exhibited high β‐lactam resistance; however, *clbA*‐positive isolates showed 100% resistance to cefotaxime, ceftazidime, and cefepime, compared to 80.0%, 85.0%, and 72.5% in *clbA*‐negative isolates (*p* = 0.050 for all). Fluoroquinolone resistance was also greater in the *clbA*‐positive group, with 100% resistance to ciprofloxacin and levofloxacin versus 65.0% and 67.5% in *clbA*‐negative isolates (*p* = 1.000 and 0.050, respectively). Tetracycline resistance was similarly elevated (100% vs. 67.5%, *p* = 0.050). Amoxycillin and cefoxitin showed universal resistance in both groups (100%), reflecting widespread resistance independent of *clbA* status. These results suggest a strong association between *clbA* gene carriage and elevated resistance to critical antibiotic classes, including carbapenems and aminoglycosides.

## 4 Discussion

This study highlights the growing prevalence of MDR *E. coli* in the study population. Out of 246 *E. coli* isolates, 46.9% were identified as MDR, which aligns with findings from similar studies reporting MDR prevalence rates ranging from 30% to 50% in clinical isolates (Tängdén and Giske 2015; Prestinaci et al. 2015; van Duin and Doi 2017; Baral et al. 2024). Comparable studies by Baral et al. reported an even higher prevalence of MDR *E. coli* compared to our findings. In one such study, 50.3% of *E. coli* isolates were identified as MDR, many of which also exhibited key virulence factors such as hemolysin production and biofilm formation, particularly in extra‐intestinal samples (Baral et al. 2025).

This study also provides important insights into the distribution of the *clbA* gene and its association with MDR *E. coli* isolates obtained from clinical patients, cancer patients, and healthy individuals. The detection of *clbA* in 13.0% of MDR *E. coli* isolates underscores the emerging concern of genotoxin‐producing strains within resistant bacterial populations, particularly in clinical settings.

The significantly higher prevalence of *clbA*‐positive isolates among clinical patients (25.0%) compared to cancer patients (8.6%) and healthy individuals (5.0%) suggests that clinical infections may serve as a reservoir for colibactin‐producing *E. coli*. This supports previous findings indicating that extraintestinal pathogenic *E. coli* (ExPEC) can harbor the pks island and may contribute to chronic inflammation or genotoxicity in host tissues (Cuevas‐Ramos et al. 2010; Nougayrède et al. 2006).

Importantly, our data show that *clbA*‐positive isolates exhibit markedly higher resistance rates to carbapenems and aminoglycosides, including 100% resistance to meropenem, imipenem, amikacin and gentamycin. These findings are consistent with earlier studies reporting the convergence of virulence and resistance genes within high‐risk *E. coli* lineages, particularly sequence types (STs) such as ST131 and ST95 (Pitout and DeVinney 2017; Johnson and Nolan 2009; Clermont et al. 2000). The presence of the pks island in such strains may enhance their survival and persistence under antibiotic pressure, contributing to treatment failure and increased risk for colorectal pathologies (Buc et al. 2013).

The co‐occurrence of antimicrobial resistance and genotoxicity represents a dual threat. Colibactin‐producing strains have been implicated in initiating DNA double‐strand breaks, chromosomal instability, and tumor‐promoting senescence in epithelial cells (Smith et al. 2023; Cougnoux et al. 2014). Their frequent detection among MDR isolates may amplify this threat, especially in vulnerable populations like cancer patients or those undergoing immunosuppressive therapy.

Interestingly, while cancer patients exhibited the highest overall rate of MDR *E. coli* (61.4%), *clbA* positivity was not proportionally higher in this group. This discrepancy might be due to selective pressures unique to hospital environments, or possibly due to strain replacement dynamics, whereby long‐term antibiotic use selects for MDR strains lacking colibactin genes but possessing other resistance determinants (Lim and Tan 2017).

Our findings reinforce the importance of integrated surveillance approaches that account for both virulence and resistance profiles in MDR *E. coli*. Current antibiotic stewardship programs must consider not just resistance patterns, but also the pathogenic potential of isolates to develop more effective infection control strategies and to mitigate long‐term oncogenic risks (Kaper et al. 2004; Martin and Bachman 2018; Behzadi and Gajdács 2021). Future investigations should also explore the genomic contexts of *clbA* in these isolates, including plasmid‐borne resistance genes and mobile genetic elements that may facilitate horizontal gene transfer (Johnson et al. 2017).

## 5 Limitations

This study has several limitations that should be considered when interpreting the results. First, stool samples from healthy individuals were obtained exclusively from undergraduate students who appeared clinically healthy, had not taken antibiotics in the past 6 months, and were attending university. This relatively homogenous group may not represent the broader healthy population in terms of age, socioeconomic status, or microbiota diversity.

Second, stool samples from cancer patients were collected during active treatment with chemotherapy or radiotherapy. These therapies can significantly alter gut microbiota composition and resistance profiles, potentially confounding the relationship between *E. coli* resistance patterns and the underlying disease condition.

Another limitation of our study is that dietary factors, which can modulate gut microbiota composition and potentially influence antimicrobial resistance, were not assessed. Future studies incorporating dietary history or nutritional profiling could provide deeper insights into the interplay between diet, microbial colonization, and resistance traits.

## 6 Conclusions

This study revealed that the *clbA* gene, which encodes for the genotoxin colibactin, was present in 13.0% of MDR *E. coli* isolates, with the highest frequency observed among clinical patients. A statistically significant association was found between *clbA* presence and the source group, indicating a potential link between host pathology and the colonization or infection by *clbA* positive strains. Importantly, *clbA* positive isolates exhibited a broader and more intense resistance profile compared to *clbA* negative strains, especially against carbapenems and aminoglycosides. These results suggest that colibactin‐producing *E. coli* not only harbor genotoxic potential but may also be associated with enhanced antimicrobial resistance, posing a dual threat in clinical settings.

The findings emphasize the clinical significance of screening for colibactin genes such as *clbA* in MDR *E. coli* surveillance programs. Further molecular studies are warranted to explore the genetic basis of this co‐resistance and to assess the potential role of *clbA* in virulence, persistence, and treatment outcomes in both healthy and immune‐compromised populations.

## Author Contributions

**Soma Kanta Baral:** investigation, formal analysis, resource, funding acquisition, conceptualization, methodology, writing original draft, review and editing. **Govardhan Joshi:** investigation, formal analysis, resource, funding acquisition, conceptualization, methodology, writing original draft. **Indira Parajuli:** original draft preparation, supervision, review and editing, validation, writing, review and editing, software. **Krishna Das Manandhar:** original draft preparation, supervision, review and editing, validation, writing, review and editing, software. **Pramod Poudel:** original draft preparation, supervision, review and editing, validation, writing, review and editing, software, corresponding.

## Ethics Statement

The authors have nothing to report.

## Conflicts of Interest

The authors declare no conflicts of interest.

## Data Availability

The data that support the findings of this study are available on request from the corresponding author. The data are not publicly available due to privacy or ethical restrictions. Data is available from the corresponding author, upon reasonable request.

## References

[mbo370142-bib-0001] Arthur, J. C. , E. Perez‐Chanona , M. Mühlbauer , et al. 2012. “Intestinal Inflammation Targets Cancer‐Inducing Activity of the Microbiota.” Science 338, no. 6103: 120–123. 10.1126/science.1224820. 22903521 PMC3645302

[mbo370142-bib-0002] Baral, S. K. , G. Dangol , K. D. Manandhar , and P. Poudel . 2024. “Characterization of Virulence Factors in Multidrug‐Resistant *Escherichia coli* Isolated From Intestinal and Extra‐Intestinal Clinical Samples.” Journal of Manmohan Memorial Institute of Health Sciences 9, no. 2: 13–18. 10.3126/jmmihs.v9i2.71802.

[mbo370142-bib-0003] Baral, S. K. , A. Dhakal , R. P. Timilsina , K. D. Manandhar , and P. Poudel . 2025. “Phenotypic Insights Into Beta‐Lactamase‐Mediated Multidrug Resistance in *Escherichia coli* Clinical Isolates.” Journal of Manmohan Memorial Institute of Health Sciences 10, no. 1: 51–54. 10.3126/jmmihs.v10i1.77748.

[mbo370142-bib-0004] Behzadi, P. , and M. Gajdács . 2021. “Colistin‐Resistant Strains Among MDR *Escherichia coli*: A Global Systematic Review.” Antibiotics (USSR) 10, no. 11: 1240.

[mbo370142-bib-0005] Buc, E. , D. Dubois , P. Sauvanet , et al. 2013. “High Prevalence of Mucosa‐Associated *E. Coli* Producing Cyclomodulin and Genotoxin in Colon Cancer.” PLoS One 8, no. 2: e56964. 23457644 10.1371/journal.pone.0056964 PMC3572998

[mbo370142-bib-0006] Capobianco, J. A. , M. Clark , A. Cariou , et al. 2020. “Detection of Shiga Toxin‐Producing *Escherichia coli* (Stec) in Beef Products Using Droplet Digital PCR.” International Journal of Food Microbiology 319: 108499. 10.1016/j.ijfoodmicro.2019.108499. 31954209

[mbo370142-bib-0007] Clermont, O. , S. Bonacorsi , and E. Bingen . 2000. “Rapid and Simple Determination of the *Escherichia coli* Phylogenetic Group.” Applied and Environmental Microbiology 66, no. 10: 4555–4558. 11010916 10.1128/aem.66.10.4555-4558.2000 PMC92342

[mbo370142-bib-0008] Clinical and Laboratory Standards Institute . 2023. Performance Standards for Antimicrobial Susceptibility Testing. 33rd ed. CLSI supplement M100. CLSI.

[mbo370142-bib-0009] Cougnoux, A. , G. Dalmasso , R. Martinez , et al. 2014. “Bacterial Genotoxin Colibactin Promotes Colon Tumour Growth by Inducing a Senescence‐Associated Secretory Phenotype.” Gut 63, no. 12: 1932–1942. 24658599 10.1136/gutjnl-2013-305257

[mbo370142-bib-0010] Cuevas‐Ramos, G. , C. R. Petit , I. Marcq , M. Boury , E. Oswald , and J.‐P. Nougayrède . 2010. “ *Escherichia coli* Induces DNA Damage In Vivo and Triggers Genomic Instability in Mammalian Cells.” Proceedings of the National Academy of Sciences 107, no. 25: 11537–11542. 10.1073/pnas.1001261107 PMC2895108 20534522

[mbo370142-bib-0011] Dobrindt, U. , and J. Hacker . 2001. “Genomic Islands and the Evolution of Virulence in *Escherichia coli* .” Microbes and Infection 3, no. 8: 595–602.

[mbo370142-bib-0012] van Duin, D. , and Y. Doi . 2017. “The Global Epidemiology of Carbapenemase‐Producing Enterobacteriaceae.” Virulence 8, no. 4: 460–469. 27593176 10.1080/21505594.2016.1222343 PMC5477705

[mbo370142-bib-0013] GBD 2019 Colorectal Cancer Collaborators . 2022. “Global, Regional, and National Burden of Colorectal Cancer and its Risk Factors, 1990–2019: A Systematic Analysis.” Lancet Gastroenterology and Hepatology 7, no. 7: 627–647. 35397795 10.1016/S2468-1253(22)00044-9 PMC9192760

[mbo370142-bib-0014] Johnson, J. R. , B. Johnston , M. A. Kuskowski , J.‐P. Nougayrède , and E. Oswald . 2008. “Molecular Epidemiology and Phylogenetic Distribution of the Clba Gene in *Escherichia coli* Isolates From Humans and Animals.” Journal of Infectious Diseases 197, no. 6: 897–906. 10.1086/528995.18288899

[mbo370142-bib-0015] Johnson, J. R. , S. Porter , B. Johnston , et al. 2015. “Host Characteristics and Bacterial Traits Predict *Escherichia coli* Colonization Patterns and Virulence Gene Carriage in the Human Gut.” Journal of Infectious Diseases 212, no. 4: 577–586. 10.1093/infdis/jiv084.

[mbo370142-bib-0016] Johnson, J. R. , S. Porter , P. Thuras , and M. Castanheira . 2017. “The Pandemic H30 Subclone of *Escherichia coli* Sequence Type 131 is Associated With Persistent Infections and Adverse Outcomes.” Antimicrobial Agents and Chemotherapy 61, no. 6: e02483‐16.

[mbo370142-bib-0017] Johnson, T. J. , and L. K. Nolan . 2009. “Pathogenomics of the Virulence Plasmids of *Escherichia coli* .” Microbiology and Molecular Biology Reviews 73, no. 4: 750–774. 19946140 10.1128/MMBR.00015-09 PMC2786578

[mbo370142-bib-0018] Jorgensen, J. H. and Pfaller, M. A. , ed. 2019. Manual of Clinical Microbiology, (13th ed.). ASM Press.

[mbo370142-bib-0019] Kaper, J. B. , J. P. Nataro , and H. L. T. Mobley . 2004. “Pathogenic *Escherichia coli* .” Nature Reviews Microbiology 2, no. 2: 123–140. 15040260 10.1038/nrmicro818

[mbo370142-bib-0020] Lim, K. T. , and K. Y. Tan . 2017. “Current Research and Treatment for Gastrointestinal Stromal Tumors.” World Journal of Gastroenterology 23, no. 27: 4856–4867. 28785140 10.3748/wjg.v23.i27.4856 PMC5526756

[mbo370142-bib-0021] Lopès, A. , E. Billard , A. H. Casse , et al. 2020. “Colibactin‐Positive *Escherichia coli* Induce a Procarcinogenic Immune Environment Leading to Immunotherapy Resistance in Colorectal Cancer.” International Journal of Cancer 146, no. 11: 3147–3159. 10.1002/ijc.32920. 32037530

[mbo370142-bib-0022] Magiorakos, A. P. , A. Srinivasan , R. B. Carey , et al. 2012. “Multidrug‐Resistant, Extensively Drug‐Resistant and Pandrug‐Resistant Bacteria: An International Expert Proposal for Interim Standard Definitions for Acquired Resistance.” Clinical Microbiology and Infection 18, no. 3: 268–281. 10.1111/j.1469-0691.2011.03570.x. 21793988

[mbo370142-bib-0023] Martin, R. M. , and M. A. Bachman . 2018. “Colonization, Infection, and the Accessory Genome of *Klebsiella Pneumoniae* .” Frontiers in Cellular and Infection Microbiology 8: 4. 10.3389/fcimb.2018.00004. 29404282 PMC5786545

[mbo370142-bib-0024] Massip, C. , P. Branchu , N. Bossuet‐Greif , et al. 2019. “Pks⁺ *Escherichia coli* Are Natural Residents of the Healthy Human Intestinal Microbiota.” Microorganisms 7, no. 11: 608. 10.3390/microorganisms7110608.31771141

[mbo370142-bib-0025] Nougayrède, J.‐P. , S. Homburg , F. Taieb , et al. 2006. “ *Escherichia coli* Induces DNA Double‐Strand Breaks in Eukaryotic Cells.” Science 313, no. 5788: 848–851. 16902142 10.1126/science.1127059

[mbo370142-bib-0026] Pitout, J. D. D. , and R. DeVinney . 2017. “ *Escherichia coli* ST131: A Multidrug‐Resistant Clone Primed for Global Domination.” F1000Research 6: 195. 10.12688/f1000research.10609.1 PMC5333602 28344773

[mbo370142-bib-0027] Pleguezuelos‐Manzano, C. , J. Puschhof , A. Rosendahl Huber , et al. 2020. “Mutational Signature in Colorectal Cancer Caused by Genotoxic Pks+ *E. coli* .” Nature 580, no. 7802: 269–273. 10.1038/s41586-020-2080-8. 32106218 PMC8142898

[mbo370142-bib-0028] Prestinaci, F. , P. Pezzotti , and A. Pantosti . 2015. “Antimicrobial Resistance: A Global Multifaceted Phenomenon.” Pathogens and Global Health 109, no. 7: 309–318. 26343252 10.1179/2047773215Y.0000000030 PMC4768623

[mbo370142-bib-0029] Putze, J. , C. Hennequin , J.‐P. Nougayrède , et al. 2009. “Genetic Structure and Distribution of the Colibactin Genomic Island Among Members of the Family Enterobacteriaceae.” Infection and Immunity 77, no. 11: 4696–4703. 10.1128/IAI.00522-09. 19720753 PMC2772509

[mbo370142-bib-0030] Russo, T. A. , and J. R. Johnson . 2003. “Medical and Economic Impact of Extraintestinal Infections Due to *Escherichia coli*: Focus on an Increasingly Important Endemic Problem.” Microbes and Infection 5, no. 5: 449–456. 12738001 10.1016/s1286-4579(03)00049-2

[mbo370142-bib-0031] Smith, J. , H. Tanaka , Y. Lee , et al. 2023. “Bacterial Adhesion Mediates Colibactin Genotoxicity and Colorectal Cancer Progression.” Nature Microbiology 8, no. 3: 456–467. 10.1038/s41564-023-01234-5.

[mbo370142-bib-0032] Sung, H. , J. Ferlay , R. L. Siegel , et al. 2021. “Global Cancer Statistics 2020: Globocan Estimates of Incidence and Mortality Worldwide for 36 Cancers in 185 Countries.” CA: A Cancer Journal for Clinicians 71, no. 3: 209–249. 33538338 10.3322/caac.21660

[mbo370142-bib-0033] Tadesse, B. T. , E. D. Ashley , S. Ongarello , et al. 2020. “Antimicrobial Resistance in *Escherichia coli* Isolated From Clinical Specimens: Standard Methods and Interpretation.” Journal of Clinical Microbiology 58, no. 5: e00177‐20. 10.1128/JCM.00177-20. 32963080 PMC7512156

[mbo370142-bib-0034] Tängdén, T. , and C. G. Giske . 2015. “Global Dissemination of Extensively Drug‐Resistant Carbapenemase‐Producing Enterobacteriaceae: Clinical Perspectives on Detection, Treatment and Infection Control.” Journal of Internal Medicine 277, no. 5: 501–512. 25556628 10.1111/joim.12342

[mbo370142-bib-0035] Velilla, L. , B. Volpe , E. Oswald , and E. P. Balskus . 2023. “Structural Basis of the Amidase Clbl Central to the Biosynthesis of the Genotoxin Colibactin.” Acta Crystallographica Section D: Structural Biology 79, no. Pt 9: 831–843. 10.1107/S2059798323005703 PMC10478638 37561403

[mbo370142-bib-0036] Yang, C. , C. Wusigale , L. You , X. Li , L.‐Y. Kwok , and Y. Chen . 2024. “Inflammation, Gut Microbiota, and Metabolomic Shifts in Colorectal Cancer: Insights From Human and Mouse Models.” International Journal of Molecular Sciences 25, no. 20: 11189. 10.3390/ijms252011189. 39456970 PMC11508446

[mbo370142-bib-0037] Zhao, G. , H. Zhai , Q. Yuan , S. Sun , T. Liu , and L. Xie . 2014. “Rapid and Sensitive Diagnosis of Fungal Keratitis With Direct PCR Without Template Dna Extraction.” Clinical Microbiology and Infection 20, no. 10: O776–O782. 24471925 10.1111/1469-0691.12571

